# TREM-1 expression in craniopharyngioma and Rathke's cleft cyst: its possible implication for controversial pathology

**DOI:** 10.18632/oncotarget.10501

**Published:** 2016-07-08

**Authors:** Yi Liu, Chao-hu Wang, Dan-ling Li, Shi-chao Zhang, Yu-ping Peng, Jun-xiang Peng, Ye Song, Song-tao Qi, Jun Pan

**Affiliations:** ^1^ Department of Neurosurgery, Nanfang Hospital of Southern Medical University, Guangzhou, China; ^2^ Department of Biometric, College of Public Health of Southern Medical University, Guangzhou, Guangdong, China

**Keywords:** craniopharyngioma, metaplasia, Rathke's cleft cyst, SE, TREM-1

## Abstract

Whether a mixed type of craniopharyngioma (CP) exists and whether papillary craniopharyngioma (pCP) is on a histopathological continuum with Rathke's cleft cyst (RCC) remain controversial. Herein, we examined the expression and localization of β-catenin, BRAF p.V600E (V600E), and triggering receptor expressed on myeloid cells-1 (TREM-1) in 58 samples including 20 pCPs, 26 adamantinomatous craniopharyngiomas (aCP), and 12 RCCs. Five aCPs were diagnosed with mixed type CPs and the remaining 21 cases were pure aCPs. Four of the 12 RCCs presented with significant squamous epithelium (SE). V600E immunoreactivity was observed in all pCPs in the cytoplasm, but not in the nuclei. aCPs and RCCs, including mixed type CP, did not express V600E. Nuclear β-catenin translocation was detected exclusively in aCPs. TREM-1 was expressed in pCPs. Additionally, TREM-1 expression was detected in the SE of 5 “mixed type” CPs, while it was absent in pure aCPs. TREM-1 was expressed in 4 RCCs with SE, but not in the remaining 8 RCCs. *TREM-1* mRNA levels were compared in cultured pCP and aCP cells. *TREM-1* mRNA level was significantly (p < 0.001; up to 4.045 fold) higher in pCPs than in aCPs. Western blotting revealed a significantly (p < 0.001; up to 7.19 fold) lower level of TREM-1 expression in aCP cells compared to that in pCP cells. Our findings further supported that RCC and pCP may represent two ends of a morphological spectrum. A variant showing overlapping histological features of aCP and pCP should not be considered as a mixed type.

## INTRODUCTION

Craniopharyngiomas (CPs) are complex epithelial neoplasms of the sellar region arising along the site of the vestigial craniopharyngeal tract. In their pure form, adamantinomatous craniopharyngiomas (aCP) and papillary craniopharyngioma (pCP) are clinicopathologically distinct [1–4]. The observation of some overlapping features, mainly the squamous epithelium (SE) component, between aCP and pCP led to the hypothesis that a mixed type may exist [5–8]. Previous reports suggest that a mixed type presenting with the characteristic histopathologic findings of aCP and pCP exists. Additionally, there are frequent admixtures of both squamous and adamantinomatous areas within the same tumor, which were only identified by conventional microscopy without immunohistochemistry. pCP's resemblance with Rathke's cleft cyst (RCC) with SE also supports the hypothesis that pCP is on a histopathological continuum with RCC [9]. However, these two hypotheses remained controversial. Some researchers consider that SE exists in aCP and RCC as a result of squamous metaplasia mediated by inflammation [10, 11].

Genetic and molecular pathologic features support a distinct pathogenesis of aCP and pCP [12–17]. It was reported that pCPs present BRAF p.V600E mutations in 95% of cases and CTNNB1 mutations are exclusive and specific to aCPs [16]. The triggering receptor expressed on myeloid cells-1 (TREM-1) is a cell surface receptor and a member of the immunoglobulin superfamily that potently amplifies inflammatory responses by inducing the secretion of pro-inflammatory mediators. TREM-1 is known as an activating receptor expressed on neutrophils, monocytes, and macrophages [18, 19]. Limited data are available on the role of TREM-1 in chronic inflammation and tumorigenesis. Previous studies [10, 11] and our studies [20, 21] showed that inflammation is common in CP and RCC. Since TREM-1 plays a significant role in the inflammatory response, whether TREM-1 is expressed in CP and RCC should be further studied.

The present study was designed to clarify two problems by using these biomarkers: one is whether aCP with SE represents a mixed type of CP; the second is whether RCC with SE is a precursor of pCP.

## RESULTS

### Patient characteristics

**Figure 1:**
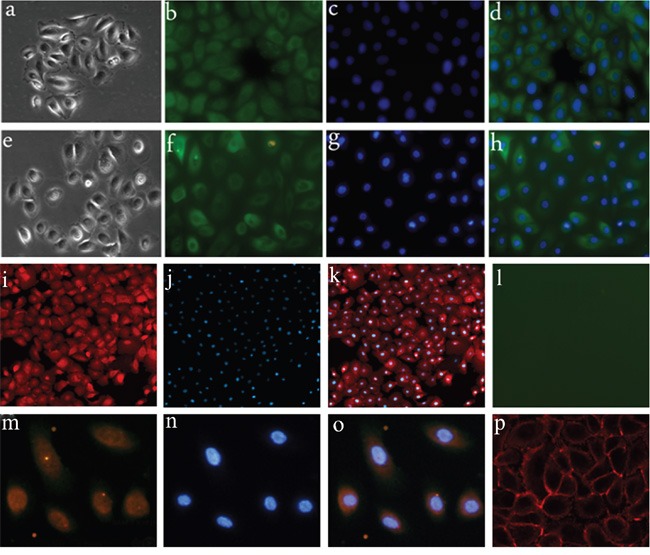
Primary cell culture of CP samples **a-d.** Primary cell culture of aCP; a. morphology of aCP cells; b-d. aCP cells are positive for pan-CK. (400×) **e-h.** Primary cell culture of pCP; e. morphology of pCP cells; f-h. pCP cells are positive for pan-CK.(400×) **i-l.** β-catenin and V600E expression in cultured aCP cells; i-k. nuclear β-catenin translocation is detected in cultured aCP cells; l. V600E immunoreactivity is absent in cultured aCP cells. (400×) **m-p.** β-catenin and V600E expression in cultured pCP cells; m-o. V600E immunoreactivity is observed in cultured pCP cells with strong detection in the cytoplasm; p. β-catenin is restricted to the cell membrane of cultured pCP cells. (400×)

Thirty three male and 25 female patients with a mean age of 37 years (range 7–61 years) were enrolled in this study. Fifty-eight samples, including 20 pCPs, 26 aCPs, and 12 RCCs, were obtained and used for analysis. Five aCP cases were diagnosed with mixed type CP according to previous studies [7, 8] and the remaining 21 cases were pure aCPs. Four of the 12 RCCs presented with significant SE. In order to eliminate the influence of mesenchymal cells in tumor tissues, we used both tumor tissues and cultured CP cells in this study. All CP samples were successfully cultured. We used the anchorage velocity-dependent separation method to exclude mesenchymal cells and passage 3-cells were used for experiments. Cultured CP cells were identified by assessing the expression of pan-CK. We also identified the cells by immunostaining using BRAF V600E and β-catenin antibodies (Figure 1).

### Differential distribution pattern of BRAF V600E in CPs and RCCs tissues

**Figure 2:**
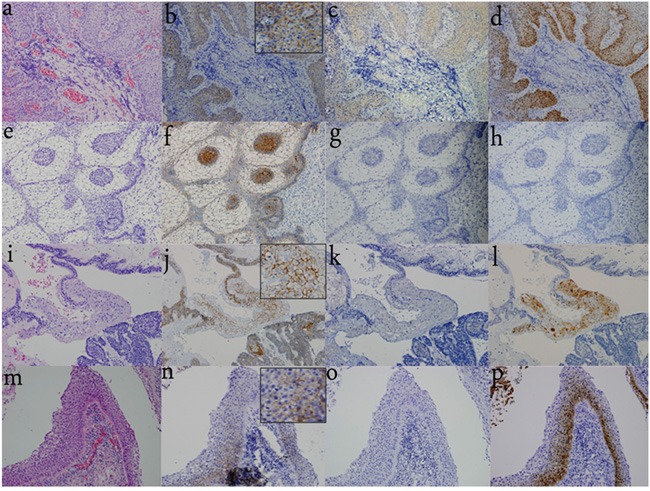
Differential distribution pattern of β-catenin, V600E, and TREM-1 in CPs and RCCs **a.** H&E staining of pCP indicates the squamous and well-differentiated, non-keratinizing epithelium. (200×) **b.** Nuclear β-catenin translocation is not observed and β-catenin expression is restricted to the cell membrane. (200×) **c.** V600E immunoreactivity is observed in the SE of pCPs with strong detection in the cytoplasm, while it is not detected in the nuclei. (200×) **d.** TREM-1 immunoreactivity is strongly detected in the cytoplasm and nuclei of the SE in all pCPs. (200×) **e.** H&E staining of pure aCP shows the classical appearance with peripheral palisading epithelium, loose ‘stellate reticulum’, and whorl-like cells. (200×) **f.** β-catenin translocation is observed especially in whorl-like cells. (200×) **g.** V600E immunoreactivity is not detected in pure aCP tissues. (200×) **h.** TREM-1 immunoreactivity is also absent in pure aCP tissues. (200×) **i.** H&E staining of “mixed type CPs” shows both SE and peripheral palisading epithelium, whorl-like cells are also detected. (200×) **j.** β-catenin translocation is observed in whorl-like cells and is restricted to the cell membrane in SE of “mixed type CP”. (200×) **k.** V600E immunoreactivity is absent in SE and other components of “mixed type CP”. (200×) **l.** TREM-1 immunoreactivity is observed in the SE of “mixed type CP” and absent in other components of “mixed type CPs”. (200×) **m.** H&E staining of RCC with stratified SE. (400×) **n.** No nuclear β-catenin translocation is observed and β-catenin expression is restricted to the cell membrane. (400×) **o.** V600E immunoreactivity is absent in SE. (400×) **p.** TREM-1 immunoreactivity is observed in the SE. (400×)

We examined the immunohistochemical distribution pattern of BRAF V600E (V600E) in all specimens. V600E immunoreactivity was strongly detected in the SE of all pCPs in the cytoplasm, but the immunoreactivity was not detected in the nuclei. V600E immunoreactivity was absent in all aCPs (including “mixed type CP”) and RCCs (including those with SE specimens) (Figure 2).

### Differential distribution pattern of β-catenin in CP and RCC tissues

Nuclear β-catenin translocation was exclusively detected in all aCPs (including “mixed type CP”), especially in epithelial nodules. β-catenin remained at the cell membrane in pCPs and RCCs (Figure 2).

### Differential distribution pattern of TREM-1 in CP and RCC tissues

TREM-1 immunoreactivity was observed in the SE of all pCPs with strong detection in the cytoplasm and nuclei. Immunoreactivity was also detected in the SE of aCPs (mixed type CP), while absent in aCPs without SE (pure aCPs). TREM-1 was expressed in 4 RCC with SE, mainly in the cytoplasm and nuclei of the SE in RCC. The remaining 8 RCCs without SE did not express TREM-1 (Figure 2).

### Mutation distributions

#### BRAF mutation

BRAF mutation was detected in all pCP patients. All of them presented with the V600E mutation. In patients with aCP, including 21 pure aCPs and 5 “mixed type” CPs, BRAF mutation was absent. All patients with RCC were also negative for BRAF mutation.

#### CTNNB1 exon 3 mutation

CTNNB1 exon 3 mutation was detected in all patients with aCP, including 21 pure aCPs and 5 “mixed type” CPs, while the mutation was absent in all patients with pCP and RCC. We found 5 mutational sites. The mutational analysis of CTNNB1 exon 3 mutations in patients with aCP is summarized in Table 1. More than 50% of the mutations (14/26) were at the “hotspot” Ser and Thr residues.

**Table 1:** Summary of the mutational analysis of CTNNB1 exon 3 in aCP

	Histological diagnosis	mutational site	Nucleotide change
1	Pure aCP	p.S37F(c.110C>T)	GGA ATC CAT TCT GGT GCC ACT > GGA ATC CAT TTT GGT GCC ACT
2	Pure aCP	p.D32G(c.95A>G)	TCT TAC CTG GAC TCT GGA ATC > TCT TAC CTG GGC TCT GGA ATC
3	Pure aCP	p.S37F(c.110C>T)	GGA ATC CAT TCT GGT GCC ACT > GGA ATC CAT TTT GGT GCC ACT
4	Pure aCP	p.S37F(c.110C>T)	GGA ATC CAT TCT GGT GCC ACT > GGA ATC CAT TTT GGT GCC ACT
5	Pure aCP	p.D32G(c.95A>G)	TCT TAC CTG GAC TCT GGA ATC > TCT TAC CTG GGC TCT GGA ATC
6	Pure aCP	p.D32G(c.95A>G)	TCT TAC CTG GAC TCT GGA ATC > TCT TAC CTG GGC TCT GGA ATC
7	Pure aCP	p.S37F(c.110C>T)	GGA ATC CAT TCT GGT GCC ACT > GGA ATC CAT TTT GGT GCC ACT
8	Pure aCP	p.T41I(c.122C>T)	GGT GCC ACT ACC ACA GCT CCT > GGT GCC ACT ATC ACA GCT CCT
9	Pure aCP	p.D32Y(c.94G>T)	TCT TAC CTG GAC TCT GGA ATC > TCT TAC CTG TAC TCT GGA ATC
10	Pure aCP	p.T41I(c.122C>T)	GGT GCC ACT ACC ACA GCT CCT > GGT GCC ACT ATC ACA GCT CCT
11	Pure aCP	p.S37F(c.110C>T)	GGA ATC CAT TCT GGT GCC ACT > GGA ATC CAT TTT GGT GCC ACT
12	Pure aCP	p.D32G(c.95A>G)	TCT TAC CTG GAC TCT GGA ATC > TCT TAC CTG GGC TCT GGA ATC
13	Pure aCP	p.D32Y(c.94G>T)	TCT TAC CTG GAC TCT GGA ATC > TCT TAC CTG TAC TCT GGA ATC
14	Pure aCP	p.D32G(c.95A>G)	TCT TAC CTG GAC TCT GGA ATC > TCT TAC CTG GGC TCT GGA ATC
15	Pure aCP	p.D32Y(c.94G>T)	TCT TAC CTG GAC TCT GGA ATC > TCT TAC CTG TAC TCT GGA ATC
16	Pure aCP	p.D32G(c.95A>G)	TCT TAC CTG GAC TCT GGA ATC > TCT TAC CTG GGC TCT GGA ATC
17	Pure aCP	p.S37C(c.110C>G)	GGA ATC CAT TCT GGT GCC ACT > GGA ATC CAT TGT GGT GCC ACT
18	Pure aCP	p.S37F(c.110C>T)	GGA ATC CAT TCT GGT GCC ACT > GGA ATC CAT TTT GGT GCC ACT
19	Pure aCP	p.T41I(c.122C>T)	GGT GCC ACT ACC ACA GCT CCT > GGT GCC ACT ATC ACA GCT CCT
20	Pure aCP	p.D32Y(c.94G>T)	TCT TAC CTG GAC TCT GGA ATC > TCT TAC CTG TAC TCT GGA ATC
21	Pure aCP	p.T41I(c.122C>T)	GGT GCC ACT ACC ACA GCT CCT > GGT GCC ACT ATC ACA GCT CCT
22	Mixed CP	p.S37C(c.110C>G)	GGA ATC CAT TCT GGT GCC ACT > GGA ATC CAT TGT GGT GCC ACT
23	Mixed CP	p.D32Y(c.94G>T)	TCT TAC CTG GAC TCT GGA ATC > TCT TAC CTG TAC TCT GGA ATC
24	Mixed CP	p.S37C(c.110C>G)	GGA ATC CAT TCT GGT GCC ACT > GGA ATC CAT TGT GGT GCC ACT
25	Mixed CP	p.T41I(c.122C>T)	GGT GCC ACT ACC ACA GCT CCT > GGT GCC ACT ATC ACA GCT CCT
26	Mixed CP	p.D32Y(c.94G>T)	TCT TAC CTG GAC TCT GGA ATC > TCT TAC CTG TAC TCT GGA ATC

### TREM-1 expression in CP cells

**Figure 3:**
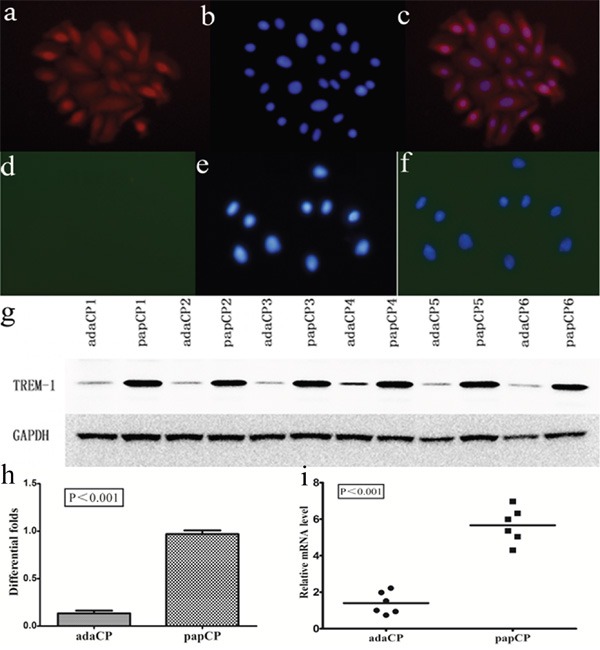
TREM-1 expression pattern in aCP and pCP cells **a-c.** TREM-1 expression is strongly detected in the cytoplasm and nuclei of pCP cells. (400×) **d-f.** TREM-1 expression is absent in aCP cells. (400×) **g-h.** Western blotting shows the protein level of TREM-1 in cultured CP cells. Evaluation of the relative TREM-1 expression revealed a significantly (p < 0.001; up to 7.19 fold) lower level in aCP (mean = 0.13 ± 0.07) compared to that of the pCP variant (mean = 0.97 ± 0.03) **i.** *TREM-1* mRNA levels of aCP and pCP cells were compared by qRT-PCR. TREM-1 relative expression level was significantly (p < 0.001; up to 4.045 fold) higher in pCP (mean = 5.66 ± 0.39) compared to that of the aCP variant (mean = 1.40 ± 0.25).

We compared TREM-1 expression in aCP and pCP cells using double-immunofluorescence staining (Figure 3). *TREM-1* mRNA levels of aCP and pCP cells were compared by quantitative reverse transcription-polymerase chain reaction (qRT-PCR) from a collection of native tumor samples comprising 6 pCP and 6 aCP cultured cells. The evaluation of the relative *TREM-1* expression revealed a significantly (p < 0.001; up to 4.045 fold) higher level in pCP cells (mean = 5.66 ± 0.39) than in aCP cells (mean = 1.40 ± 0.25). Western blotting was employed to assess TREM-1 protein level in the same CP cultured cells. TREM-1 expression levels were significantly (p < 0.001; up to 7.19 fold) lower in aCP cells (mean = 0.13 ± 0.07) than in pCP cells (mean = 0.97 ± 0.03) (Figure 3).

### TREM-1 expression in CP tissues

**Figure 4:**
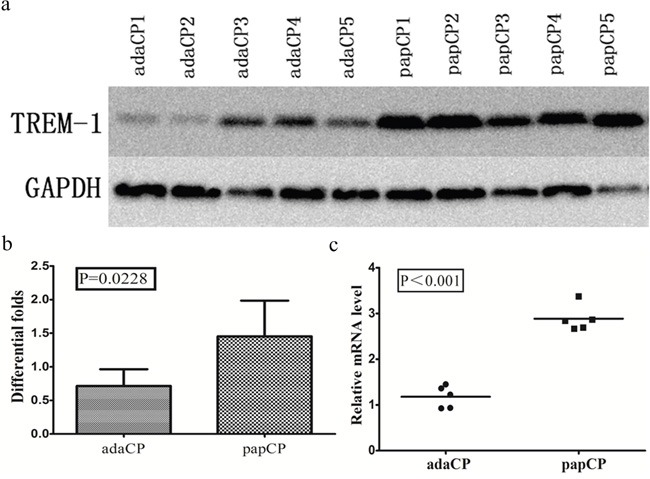
TREM-1 expression pattern in aCP and pCP tissues **a-b.** Western blotting shows the protein level of TREM-1 in CP tissues. TREM-1 expression level is significantly (p = 0.0228; up to 1.89 fold) lower in aCP tissues (mean = 0.714 ±0.112) compared to that of the pCP variant (mean = 1.453 ± 0.238). **c.** Evaluation of the relative TREM-1 expression revealed a significantly (p < 0.001; up to 2.08 fold) higher level in pCP (mean = 2.99 ± 0.12) compared to that of the aCP tissues (mean = 1.28 ± 0.11)

TREM-1 levels in aCP and pCP tissues were also compared by western blotting and qPCR from the same 6 pCP and 6 aCP samples. Unfortunately, one aCP and one pCP sample were too small for western blotting. Thus, only 5 aCP and 5 pCP samples were used. TREM-1 expression levels were significantly (p = 0.0228; up to 1.89 fold) lower in aCP tissues (mean = 0.714 ± 0.112) than that in pCP tissues (mean = 1.453 ± 0.238), as seen by western blotting. *TREM-1* mRNA levels of the same 5 aCP and 5 pCP tissues samples were compared by qRT-PCR. *TREM-1* expression levels were significantly (p < 0.001; up to 2.08 fold) higher in pCP tissues (mean = 2.99 ± 0.12) than in aCP tissues (mean = 1.28 ± 0.11) (Figure 4).

### Differential distribution pattern of TREM-1 in SE of other tissues

**Figure 5:**
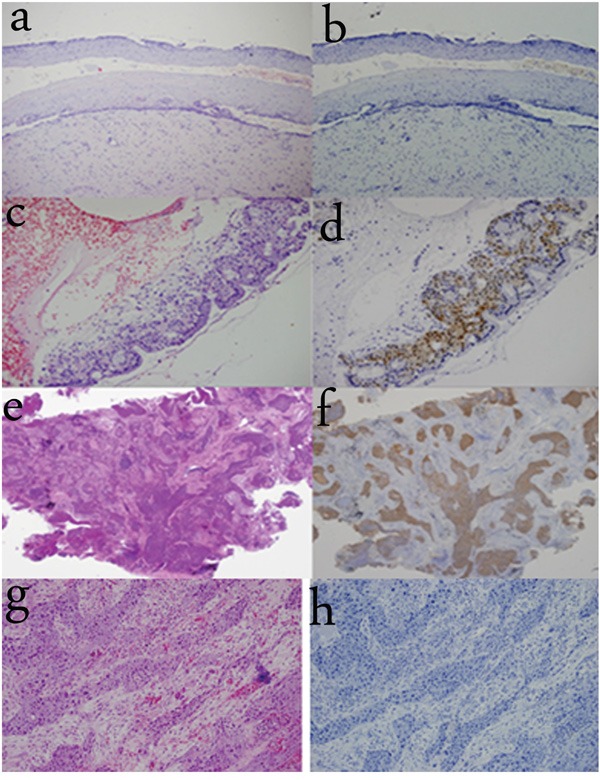
TREM-1 expression in other SE tissues **a-b.** TREM-1 immunoreactivity is absent in normal cervical squamous epithelium. (100×) **c-d.** TREM-1 immunoreactivity is observed in metaplastic cervical squamous epithelium and strongly detected in the cytoplasm and nuclei. (200×) **e-f.** TREM-1 immunoreactivity is observed in squamous cell lung cancer (originating from metaplastic SE). (40×) **g-h.** TREM-1 immunoreactivity is absent in tongue carcinoma (originating from SE). (200×)

In order to examine the significance of TREM-1 expression in distinguishing normal SE from metaplastic SE, we examined TREM-1 expression in various tissues, including cervical SE and metaplastic SE from cervicitis. TREM-1 expression was strongly detected in the cytoplasm and nuclei of metaplastic cervical SE, while absent in normal cervical SE. We also examined TREM-1 expression in tumor tissues, including tongue carcinoma (originating from SE) and squamous cell lung cancer (originating from metaplastic SE). TREM-1 was strongly expressed in the cytoplasm and nuclei in squamous cell lung cancer, while absent in tongue carcinoma (Figure 5).

## DISCUSSION

CP and RCC are the most frequent parasellar epithelial lesions. Previous studies successfully described distinct pathologic features of CP and RCC, which are valuable for differential diagnosis. However, some debates remain as to whether there is a disease spectrum extending from RCC to pCP and whether a mixed histological pattern of CP exists [9–11].

A significant confusion may exist between the diagnosis of RCC and craniopharyngioma given that there may be overlapping histological features. Rathke's cleft cysts are classically lined by a simple cuboidal or columnar epithelium. Some RCCs appear to be a smooth transition from ciliated columnar epithelium to ciliated SE, to the stratified SE of craniopharyngiomas, which led to the hypothesis that the basal cells of RCC may transform to papillary-type craniopharyngiomas following squamous metaplasia. However, other researchers attributed focal squamous metaplasia in RCC to chronic inflammation in the cyst walls [4, 9].

The BRAF V600E mutation was recently described as a genetic hallmark of papillary craniopharyngiomas. Buslei *et al*. analyzed the BRAF mutational status of 33 Rathke's cleft cyst and 18 papillary craniopharyngioma specimens by immunohistochemistry using a monoclonal antibody (V600E) that selectively recognizes the BRAF V600E mutant epitope and additional BRAF pyrosequencing. All control cases of papillary craniopharyngiomas showed evidence of BRAF V600E mutation either by immunohistochemistry or by pyrosequencing. Three out of 33 cases with Rathke's cleft cysts showed genetic BRAF V600E alterations. Histological re-evaluation indicated that two of three positive cases were papillary craniopharyngiomas and the third case presented with unclear histology [22]. Our results were similar. In fact, immunohistochemistry indicated that SE from RCC was also BRAF V600E negative. The results may indicate that there is no disease spectrum extending from RCC to pCP.

Both pCP and RCC SE were immunoreactive for TREM-1. Moreover, TREM-1 was expressed in squamous metaplasia of cervicitis and lung cancer, while normal cervical SE and tongue carcinoma did not express TREM-1. The results indicated that TREM-1 may be a novel marker of squamous metaplasia. The metaplastic theory posits that differentiated SE that forms part of the anterior pituitary or pituitary stalk undergoes metaplastic transformation to form pCP [10]. Thus, the fact that pCP epithelium was positive for TREM-1 was expected. Negative BRAF V600E immunoreactivity and positive TREM-1 immunoreactivity in RCC SE also support that there is no disease spectrum extending from RCC to pCP and the SE in RCC should be considered as metaplastic. Ogawa *et al*. demonstrated that patients with RCC presenting with significant squamous cell metaplasia had an extremely high risk (up to 50%) of postoperative re-enlargement and often necessitated therapeutic re-intervention [23]. Our results were similar to those of our previous study [21], while results from Buslei *et al.* indicate that the presence of squamous epithelial metaplasia or stratified SE in RCC is not a risk factor [22]. Duan *et al.* demonstrated that TREM-1 significantly promoted proliferation and invasion, but inhibited apoptosis of hepatocellular carcinoma (HCC) cells, which may be a prognostic factor for the clinical outcome of HCC [24]. Whether TREM-1 expression can explain why patients with RCC presenting with significant squamous metaplasia have an extremely high risk of recurrence should be further studied.

Several researchers presented evidence against such a rigid classification into aCPs and pCPs. Mixed type of craniopharyngioma was mainly defined as aCP with SE component. Petito *et al.* were among the first pathologists who questioned the validity of this distinction, after observing a high rate of lesions in a series of 245 CPs (approximately 33%) presenting a mixture of both variants [8]. In a series of 131 CPs reviewed by Szeifert *et al.*, 38% of the cases belonged to the adamantinomatous group, 26% corresponded to the squamous epithelial type, and 15% of the cases corresponded to a mixed variant combining features from both types [25]. In a series of 56 patients studied by Miller *et al.,* three cases showed a mixed histological pattern [26]. Crotty *et al.* also reported 4 cases of mixed CPs in their seminal review of the squamous-papillary type; three of these cases exhibited alternating areas with definite adamantinomatous and papillary-squamous differentiation, while the epithelium displayed an intermingled appearance in the fourth lesion [27].

TREM-1 was also expressed in all SE of pCP and SE of RCC, while absent in RCC without SE. As the SE of aCP does not harbor V600e mutation, we concluded that the SE of aCP was of a different origin than the SE of pCP, indicating that a mixed type should not exist. Since SE in aCP, pCP, and RCC expresses TREM-1, we concluded that the SE may become metaplastic.

TREM-1 was expressed in all 5 mixed type CPs (aCP with SE), while, in pure aCP, TREM-1 was not expressed. In mixed type CPs, TREM-1 was only expressed in SE, while absent in adamantinomatous areas, including the whorl-like cells and palisaded columnar epithelium. In all 5 “mixed” type CPs, V600E immunoreactivity was absent in SE and V600E mutation was not detected, indicating that SE in mixed type CP was not of the same origin as that in pCP. Thus, aCP with overlapping histological features of pCP should not be considered as a “mixed type” CP. In our previous study, inflammatory cell infiltration was common in aCP and may induce non-squamous epithelium to develop into squamous epithelial cells [28]. Positive TREM-1 immunoreactivity in SE of aCP suggests that SE in aCP may undergo metaplasia via inflammation.

Our additional findings that TREM-1 was only expressed in metaplastic SE, while absent in normal SE, suggest that TREM-1 is a novel marker of metaplasia. Based on our findings, RCC and pCP may represent two sides of a morphological spectrum, but seem to be distinct entities regarding their genetic make-up and, therefore, should be accurately differentiated. A variant showing overlapping histological features from aCP and pCP should not be considered as a mixed type. The SE in RCC and aCP may undergo metaplasia due to inflammation. However, there are some limitations in this study. First, the number of patients enrolled in this study may be too small to provide a strong conclusion. Secondly, the mechanisms involved in the genetic and molecular biological processes remain unknown. Thirdly, part of our study was similar to Buslei et al 's, however, Trem-1 expression pattern further supported Buslei et al 's result that RCC and pCP may represent two ends of a morphological spectrum. Future studies are warranted to determine the role played by TREM-1 in CP and RCC.

## MATERIALS AND METHODS

### Patients

Surgical specimens from 58 patients with CPs and RCCs were retrieved from the Neurosurgery Department of Nanfang hospital from January 2014 to December 2015. Each tumor sample was classified according to World Health Organization guidelines using hematoxylin and eosin (H&E) as well as immunohistochemical staining (e.g., pan-cytokeratin, β-catenin, and V600E). Informed consent from each patient was obtained for all specimens for further scientific investigation, as approved by the local ethics committee of Southern Medical University.

### Cell culture

In order to eliminate the influence of mesenchymal cells in CP tissues, we used cultured CP cells for our experiments. The CP specimens for tissue culture were minced into 1-mm fragments and cultured as previously reported [29–31]. We used the anchorage velocity-dependent separation method to exclude mesenchymal cells and passage 3-cells were used for experiments. The cultured CP cells were identified by assessing the expression of pan-CK. We also identified the cells by immunohistochemistry using V600E and β-catenin antibodies.

### Immunohistochemistry

Surgical samples were prepared as previously described [32]. Briefly, paraffin-embedded sections were stained. Sections were cut at 4 μm. After deparaffinization and rehydration, the sections were heated for 20 min in sodium citrate buffer (pH 6.0). For BRAF V600E, antigen retrieval was performed at pH 9, using a microwave oven. Peroxidases were blocked by incubating the sections in 3% hydrogen peroxide for 10 min. The sections were incubated overnight at 4°C with a mouse monoclonal anti-BRAF V600E (dilution 1:100, Spring Bioscience Germany, E19290), rabbit polyclonal anti-CTNNB1 (dilution 1:100, ABclonal, Wuhan, China, A2064), rabbit polyclonal anti-TREM-1 (dilution 1:150, Sigma-Aldrich, St Louis, MO, USA, HPA 005563), and mouse monoclonal anti-pan cytokeratin (dilution 1:50, Abcam, Cambridge, MA, USA, AB7753). Following washing with PBS, the slides were incubated with the 2-step Dako REALTM EnVisionTM/HRP, Rabbit/Mouse (ENV) reagent (K-5007, Dako, Glostrup, Denmark). Visualization was achieved using DAB chromogen for 30–60 seconds. The sections were counterstained using hematoxylin, dehydrated, and then mounted using permount. In the negative controls, the primary antibody was replaced with PBS. Double-immunofluorescence staining was performed manually as described elsewhere [29] Cy2-anti-mouse and Cy3-anti-rabbit (1:1000; Life Technologies, Carlsbad, CA, USA) served as fluorescent secondary antibodies. Cell nuclei were stained with DAPI (Sigma-Aldrich) for 5 min at room temperature.

### Detection of TREM-1 in CP cells by western blot

Cells were washed twice with 1× PBS and were then combined with 100 μL of RIPA lysis buffer containing 1% PMSF protease inhibitor. Cells were lysed at 4°C for 20 min and the solution was centrifuged at 12,000 rpm for 30 min. The supernatant was collected for protein assay using the bicinchoninic acid (BCA) method. Proteins were separated using 10% sodium dodecyl sulfate polyacrylamide gel electrophoresis and transferred to a polyvinylidene difluoride membrane. After blocking, the membrane was incubated at 4°C overnight with rabbit polyclonal anti-TREM-1 (dilution 1:1200, Sigma-Aldrich, HPA 005563). After washing, the secondary antibody, anti-rabbit-HRP, was used. Bands were visualized using an enhanced-chemiluminescence kit (Amersham Pharmacia Biotechnology Inc., Piscataway, NJ, USA). An anti-GAPDH antibody (dilution 1:1000, KeyGEN BioTECH, Jiangsu, China, KGAA002-2) was used as a loading control. Each experiment was performed in triplicate.

### Quantitative reverse transcription PCR

Total RNA of cultured aCP and pCP cells was isolated with Trizol Reagent (TaKaRa, Tokyo, Japan) according to manufacturer's protocol. After digestion with RNase-free DNase I (TaKaRa), the total amount of RNA was determined by measuring probes on a NanoDrop (Thermo Fisher Scientific, Waltham, MA, USA), followed by reverse transcription using SuperScript First-Strand Synthesis System (Invitrogen, Carlsbad, CA, USA) with oligo (dT) primers. qRT-PCR with Sybr Green II (Applied Biosystems, Santa Clara, CA, USA) was employed to quantitatively assess the expression of TREM-1 in 6 aCP and 6 pCP cultured cells. All analyses were carried out with the Applied Biosystems 7500 Fast Real-Time-PCR. β-actin was used as an endogenous control for cDNA amount.

Primers for human *TREM-1* were as follows: forward, 5′-GGCCACACCAACCTTCTG-3 and reverse, 5′-AGTGCCTGCCTCAATGTCTCCA-3′. Primers for actin were as follows: forward, 5′-AGAAAATCTGGCACCACACC-3′ and reverse, 5′-AGAGGCGTACAGGGATAGCA-3′. Analysis was conducted using the 2^-ΔΔCT^ method according to the manufacturer's instructions (Applied Biosystems). All analyses were carried out in quadruplicate and evaluated statistically.

### Mutation analysis of BRAF V600E and beta-catenin (CTNNB1 exon 3)

Tumor genomic DNA from each formalin-fixed and paraffin-embedded slide was extracted with the Maxwell system (Promega, Madison, WI, USA). The mutation status was analyzed with the 70plex liquidchip platform (Surexam, Guangzhou, China) for the 70 alleles. The 70plex includes five major steps: (1) multiplex PCR to amplify 70 target genes; (2) exonuclease I and shrimp alkaline phosphatase (EXOSAP) cleaning to remove excess nucleotides and primers; (3) allele specific primer extension where EXO-SAP-cleaned PCR products were amplified with 70 specific primers that were linked to 70 universal tags; (4) hybridization to beads, and (5) Luminex analysis and the median fluorescence intensity was read and analyzed. As part of quality control, samples were randomly sent to independent companies for DNA sequencing analysis.

### Statistics

Statistical analyses were performed using SPSS statistical software for Windows version 19.0 (SPSS, Chicago, IL, USA). A p value <0.05 was considered statistically significant. When the samples came from a normally distributed population, an unpaired Student *t*-test was conducted to resolve hypothesized differences.
